# CT-based radiomics combined with hematologic parameters for survival prediction in locally advanced esophageal cancer patients receiving definitive chemoradiotherapy

**DOI:** 10.1186/s13244-024-01647-2

**Published:** 2024-03-25

**Authors:** Jinfeng Cui, Dexian Zhang, Yongsheng Gao, Jinghao Duan, Lulu Wang, Li Li, Shuanghu Yuan

**Affiliations:** 1 Center for Medical Integration and Practice, Shandong University, Jinan, Shandong China; 2 Department of Pathology, Shandong Cancer Hospital and Institute, Shandong First Medical University and Shandong Academy of Medical Sciences, Jinan, Shandong China; 3 Department of Radiation Oncology and Shandong Provincial Key Laboratory of Radiation Oncology, Shandong Cancer Hospital and Institute, Affiliated to Shandong University, Shandong First Medical University and Shandong Academy of Medical Sciences, No. 440 Jiyan Road, Jinan, Shandong 250117 China; 4 Department of Oncology, The People’s Hospital of Leling, Leling, Shandong China; 5 Department of Radiation Oncology, Shandong Cancer Hospital Affiliated to Shandong University, Jinan, Shandong China; 6 Department of Radiation Oncology, The First Affiliated Hospital of USTC, Division of Life Sciences and Medicine, University of Science and Technology of China, Hefei, Anhui China

**Keywords:** Esophageal squamous cell cancer, Hematologic parameters, Nomogram, Overall survival (OS), Radiomics

## Abstract

**Objectives:**

The purpose of this study was to investigate the prognostic significance of radiomics in conjunction with hematological parameters in relation to the overall survival (OS) of individuals diagnosed with esophageal squamous cell carcinoma (ESCC) following definitive chemoradiotherapy (dCRT).

**Methods:**

In this retrospective analysis, a total of 122 patients with locally advanced ESCC were included. These patients were randomly assigned to either the training cohort (*n* = 85) or the validation cohort (*n* = 37). In the training group, the least absolute shrinkage and selection operator (LASSO) regression was utilized to choose the best radiomic features for calculating the Rad-score. To develop a nomogram model, both univariate and multivariate analyses were conducted to identify the clinical factors and hematologic parameters that could predict the OS. The performance of the predictive model was evaluated using the C-index, while the accuracy was assessed through the calibration curve.

**Results:**

The Rad-score was calculated by selecting 10 radiomic features through LASSO regression. OS was predicted independently by neutrophil-to-monocyte ratio (NMR) and Rad-score according to the results of multivariate analysis. Patients who had a Rad-score > 0.47 and an NMR > 9.76 were at a significant risk of mortality. A nomogram was constructed using the findings from the multivariate analysis. In the training cohort, the nomogram had a C-index of 0.619, while in the validation cohort, it was 0.573. The model’s accuracy was demonstrated by the calibration curve, which was excellent.

**Conclusion:**

A prognostic model utilizing radiomics and hematologic parameters was developed, enabling the prediction of OS in patients with ESCC following dCRT.

**Critical relevance statement:**

Patients with esophageal cancer who underwent definitive chemoradiotherapy may benefit from including CT radiomics in the nomogram model.

**Key points:**

• Predicting the prognosis of ESCC patients before treatment is particularly important.

• Patients with a Rad-score > 0.47 and neutrophil-to-monocyte ratio > 9.76 had a high risk of mortality.

• CT-based radiomics nomogram model could be used to predict the survival of patients.

**Graphical Abstract:**

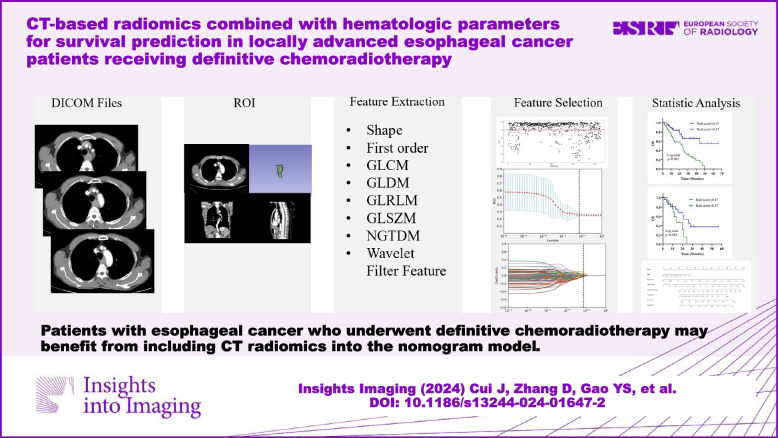

## Introduction

A recent study showed that esophageal cancer (EC) was the sixth leading cause of death worldwide, and it was the seventh most common malignancy [1]. In contrast to European and American nations, around 90% of EC cases in China consist of esophageal squamous cell carcinoma (ESCC) [2]. The majority of individuals with locally advanced ESCC have missed the chance for surgical intervention, and definitive chemoradiotherapy (dCRT) has been advised as the first-line method of treatment [3]. Nevertheless, the outcome remains poor as a result of its extremely aggressive characteristics, with a mere 23–44.7% 3-year overall survival (OS) rate [3–6]. Patients at higher risk of mortality after dCRT may benefit from more adjuvant therapy or more frequent follow-up. Therefore, it is crucial to appropriately quantify the mortality risk for each patient in order to improve OS in those diagnosed with locally advanced ESCC who are treated with dCRT.

The use of TNM staging remains a widely employed approach in clinical practice for the purpose of prognosticating patient outcomes [7]. While the clinical staging approach serves as a fundamental framework for assessing the prognosis of patients at various stages, its ability to predict survival in individuals with identical disease stages is restricted. Furthermore, some studies have provided evidence indicating that the clinical staging system lacks reliability in its ability to predict survival outcomes among individuals diagnosed with locally advanced EC [8–10].

In recent times, various clinical and imaging biomarkers, such as radiomics features and hematology-related factors, have been examined and confirmed as reliable indicators for predicting treatment response and prognosis [11–14]. Radiomics is a non-invasive tool that utilizes imaging data (such as CT, MR, and PET) to extract quantitative features in a high-throughput manner. This approach provides additional important information [15]. Research conducted by Cui et al. [16] proved that the integration of radiomics features and clinical features in a model was able to accurately predict the OS and the progression-free survival in patients with ESCC who underwent dCRT. The integrated model demonstrated superior performance in comparison to both the radiomics model and the clinical feature model. In addition to radiomics, which involves the extraction of characteristics from localized tumors, hematological indicators provide a means of assessing systemic inflammatory responses, which have been linked to the prognosis of several forms of cancer [17, 18]. Platelet-to-lymphocyte ratio (PLR) and total circulating lymphocyte count have been shown to affect prognosis in individuals with ESCC. The research found that these factors might be considered independent variables [19]. Although previous studies have separately evaluated the impact of radiomic and hematological parameters on prognosis, the combination to predict survival is still rare.

This research examined the possible predictive significance of radiomics and devised a model that integrates clinical data, radiomics, and hematological parameters to forecast OS in patients with ESCC. A validation cohort was used to test the model’s predictive performance.

## Materials and methods

### Patients

The study was approved by the ethical review committee of Shandong Cancer Hospital and Institute. Patients who received dCRT at our institution for ESCC between 2015 and 2019 were included in this retrospective analysis. Enrolled patients fulfilled the following inclusion criteria: (1) ESCC diagnosed pathologically; (2) TNM staging of II and III, clinical stage II included cT2N0-1M0 and cT3N0M0, while stage III included cT3N1M0 and cT1-3N2M0; (3) patients with complete pretreatment blood routine and clinical information; (4) availability of high-quality pretreatment contrast-enhanced CT; (5) treated with chemoradiotherapy. The radiotherapy segmentation mode was 1.8 to 2 Gy each time, once a day, five times a week. The chemotherapy regimen was paclitaxel combined with platinum, and each patient received 4–6 cycles of chemotherapy every 21 days. Patients who underwent curative surgical treatment, those who received blood transfusion within 1 week prior to treatment, and those who had been taking glucocorticoids and other medications that affect blood routine results 8 weeks prior to treatment were excluded. In addition, patients with tumors smaller than 5 mm thick or luminal obstruction less than 10 mm were excluded.

Following extensive screening, 122 patients with ESCC were randomly assigned to one of two groups, with a 7:3 split. As can be seen in Table 1, there were a total of 85 patients in the training group and 37 patients in the validation group. The AJCC 8th edition TNM classification [20] was used for the clinical staging of EC.

**Table 1:** Comparison of patients’ characteristics between training and validation groups

**Variables**		**Training group (** ***n*** ** = 85)**	**Validation group (** ***n*** ** = 37)**	***p***
Gender	Male	64 (75.3)	26 (70.3)	0.562
	Female	21 (24.7)	11 (29.7)	
Age	≤ 65	37 (43.5)	18 (48.6)	0.601
	> 65	48 (56.5)	19 (51.4)	
KPS	≤ 80	41 (48.2)	19 (51.4)	0.752
	> 80	44 (51.8)	18 (48.6)	
Smoking status	Never	43 (50.6)	18 (48.6)	0.844
	Former/current	42 (49.4)	19 (51.4)	
Alcohol consumption	Never	52 (61.2)	24 (64.9)	0.699
	Former/current	33 (38.8)	13 (35.1)	
Tumor location	Cervical	7 (8.2)	3 (8.1)	0.934
	Upper thoracic	35 (41.2)	13 (35.1)	
	Middle thoracic	29 (34.1)	14 (37.8)	
	Lower thoracic	14 (16.5)	7 (18.9)	
Clinical stage	II	12 (14.1)	7 (18.9)	0.501
	III	73 (85.9)	30 (81.1)	
Radiation dose	> 60 Gy	13 (15.3)	3 (8.1)	0.237
	> 50.4 ≤ 60 Gy	57 (67.1)	23 (62.2)	
	≤ 50.4 Gy	15 (17.6)	11 (29.7)	
Chemoradiotherapy	SCRT	44 (51.8)	16 (43.2)	0.387
	CCRT	41 (48.2)	21 (56.8)	
Radiation therapy	3D-CRT	14 (16.5)	7 (18.9)	0.742
	IMRT	71 (83.5)	30 (81.1)	

*Abbreviations: KPS* Karnofsky Performance Status, *SCRT* sequential chemoradiotherapy, *CCRT* concurrent chemoradiotherapy, *IMRT* intensity modulated radiation therapy, *3D-CRT* 3-dimensional conformal radiation therapy

### Data collection and CT images acquisition

The electronic medical record system of our institution was used to extract data pertaining to the clinical features and hematological indicators within 1 week of patients prior to their treatment. Clinical features, such as gender, age, Karnofsky Performance Status (KPS), smoking status, alcohol consumption, tumor location, clinical stage, radiation dose, and chemoradiotherapy regimens, are detailed in Table 1. The main objective of this research was OS, in which the designated time frame commences with the first day of the pathologist’s diagnosis and concludes on the ultimate day of clinical follow-up or upon the occurrence of mortality resulting from any cause. Notable hematological indicators encompass the absolute neutrophil count (ANC), the absolute platelet count (APC), the absolute monocyte count (AMC), the absolute lymphocyte count (ALC), the neutrophil to lymphocyte ratio (NLR), the neutrophil to monocyte ratio (NMR), the lymphocyte to monocyte ratio (LMR), and the PLR.

The retrieval of Digital Imaging and Communications in Medicine (DICOM) data from the Picture Archiving and Communication System (PACS) at the hospital was performed for the purpose of reconstructing the CT scans. Patients had to fast for 4 h before CT imaging. The scanning parameters of CT were as follows: section thickness 5.0 mm, tube voltage 120 kV, tube current 220 mA. Iopromide injection [21] (300 mg/mL) was rapidly injected through the patient’s elbow vein with a high-pressure syringe at a flow rate of 2 mL/s at a dose of 1.5 mL/kg body weight.

### Extraction of radiomics features and segmentation of ROI

**Fig. 1:**
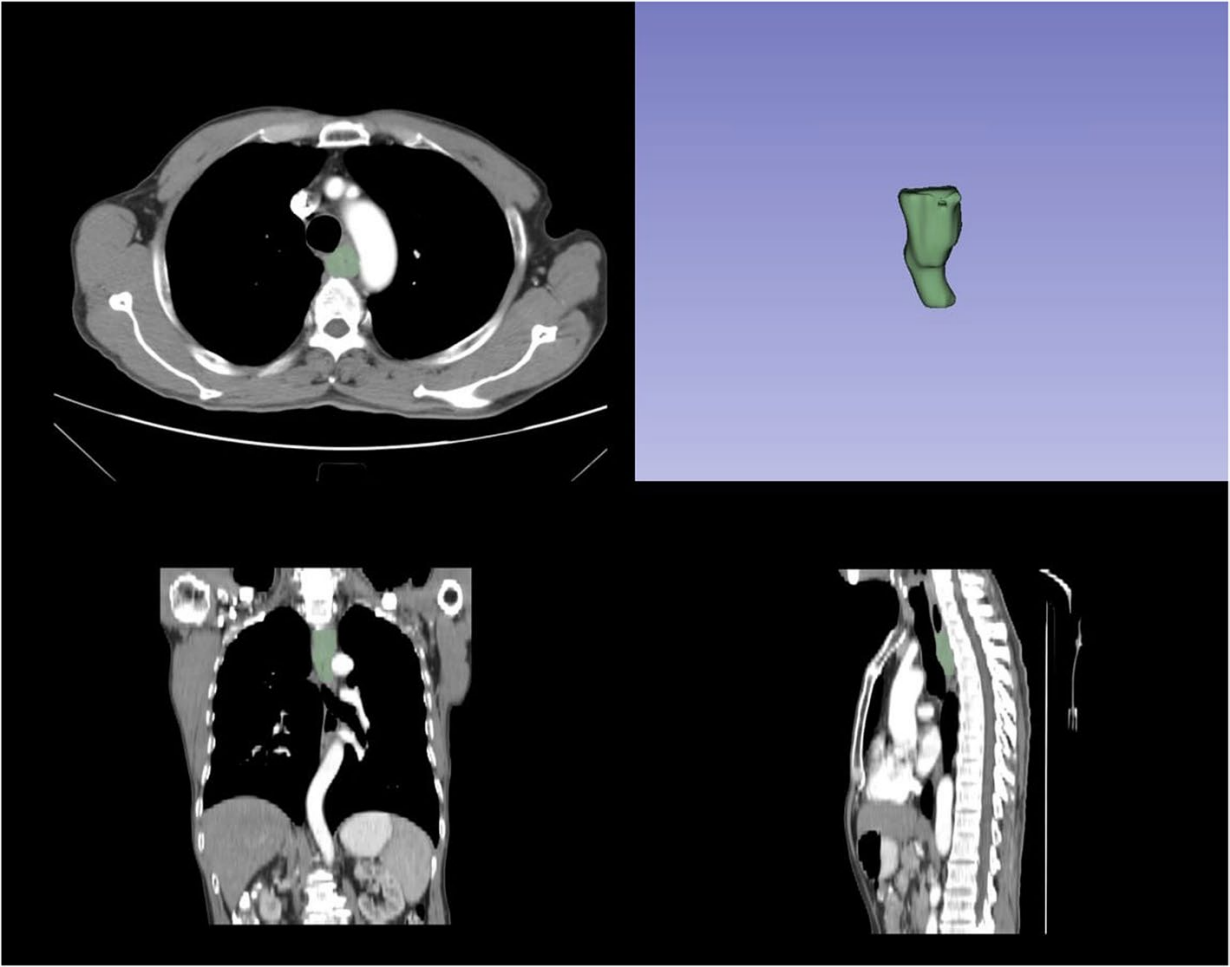
Segmentation of ROI

The original tumor was identified as the region of interest (ROI) using 3D-Slicer software (Version 4.11.0) by two radiologists with extensive clinical diagnostic experience spanning 10 years. They were blinded to the patients’ clinical and hematological information. The arterial phase of the CT imaging was used for ROI segmentation. Primary tumors with thickness > 5 mm or lumen obstruction > 10 mm were mapped layer by layer using 3D-Slicer software (Fig. 1). During this identification process, metastatic lymph nodes, nearby air, blood vessels, fat, and peripheral organs were excluded from consideration. The PyRadiomics open-source program inside the 3D-Slicer software was used to extract a total of 851 characteristics from the original tumors of every individual in the study.

### Radiomic features selection and Rad-score building

*Z*-score normalization was used to standardize each patient’s characteristics so that the data could be compared more easily. A two-stage feature selection method was implemented to reduce the likelihood of bias and overfitting caused by an abundance of features. The methods employed in this research include the use of statistical techniques such as the inter-class correlation coefficient (ICC ≥ 0.8) and the least absolute shrinkage and selection operator (LASSO) regression. Using a LASSO model and a 10-fold cross-validation strategy, the optimal value for the parameter lambda was determined. Lambda was chosen using the mean square error (MSE) as the criterion. We linearly combined the features with their corresponding weight coefficients to arrive at the Rad-score for each patient.

### Statistical analysis

SPSS 25.0 (IBM, USA) was used to do the statistical analyses. The X-tile software [22] was used to identify the best acceptable threshold for continuous data. Python software (version 3.9) was used to create the code for the LASSO regression method. Patients’ demographics and clinical characteristics were compared using the chi-square test. The OS curves were constructed using the Kaplan–Meier technique, and survival rates were compared using the log-rank test. For both the univariate and multivariate analyses, the Cox proportional hazard model was utilized. We used R (version 4.2.1) and its survivor and regression model strategies (rms) libraries to develop and validate a nomogram model. The model was evaluated using Harrell’s concordance index (c-index) and calibration curve. In order to determine whether or not a test was statistically significant, all two-tailed tests were run using a *p*-value threshold of 0.05.

## Results

### Patient characteristics

In the current research, 122 people who were diagnosed with ESCC treated with dCRT between 2015 and 2019 were analyzed retrospectively. In Table 1, we can see a breakdown of the patients’ basic information and clinical data. A total of 59 persons died after a median follow-up period of 29.5 months. The overall cohort had a median OS of 24.3 months. The subjects were split into a training cohort and a validation cohort so that a radiomics-based model could be created to predict OS. The median OS was 26.2 months in the training group and 21.2 months in the validation group. All *p* > 0.05 indicated that there were no significant differences in baseline characteristics between the two groups.

### Feature selection results and Rad-score building

Feature extraction was conducted using the PyRadiomics open-source software, which was incorporated into the 3D-Slicer application. The aforementioned methodology resulted in the generation of 851 derived features from the ROI for each unique patient. The included features consisted of 107 original features, including 18 features associated with first-order statistics, 14 features related to shape, 75 features related to texture, and 744 features derived from wavelet analysis.

**Fig. 2:**
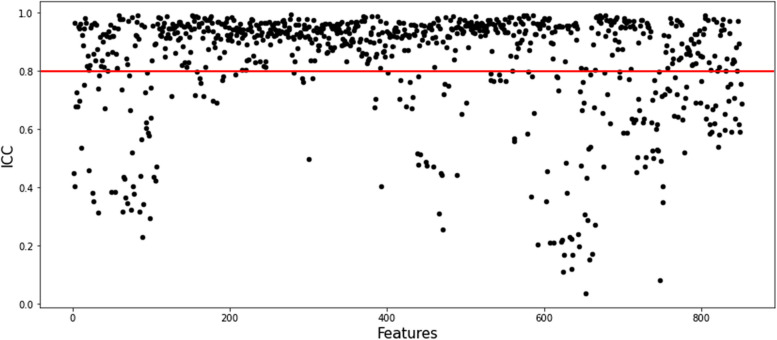
Radiomics features stability evaluation with ICC

**Fig. 3:**
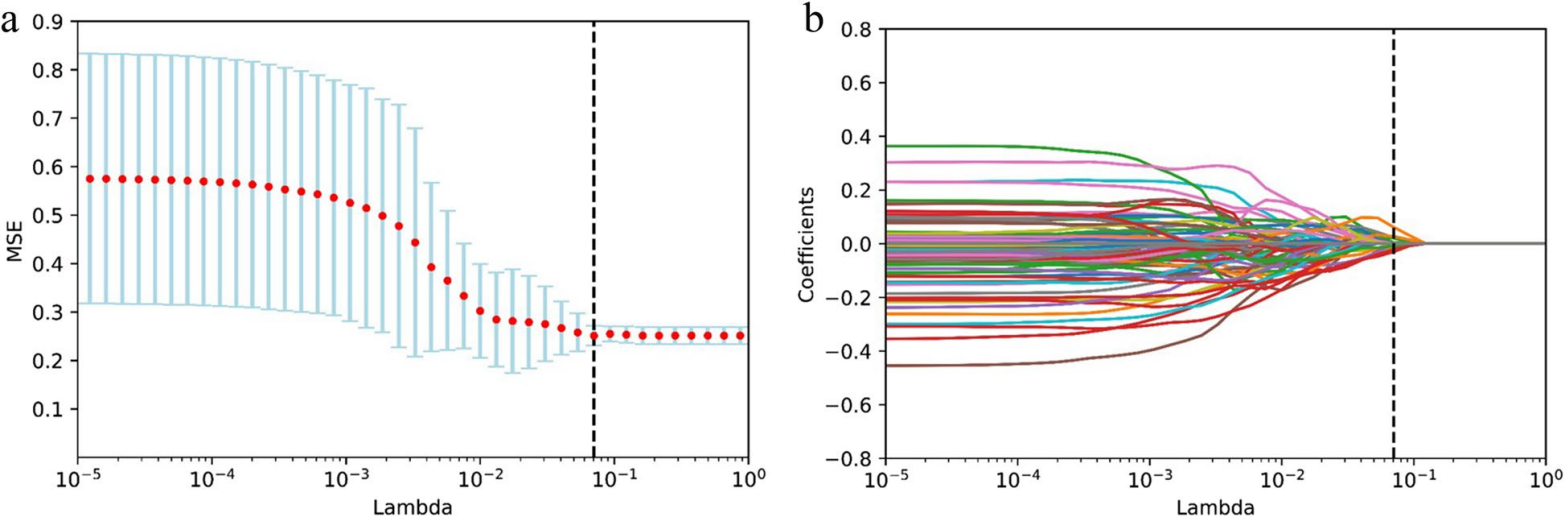
Radiomic features associated with OS were selected using LASSO regression models. **a** The cross-validation curve. The vertical axis is the mean square error, and the horizontal axis is lambda (*λ*). **b** Coefficients curves of radiomic features. The vertical axis represents the radiomic features’ coefficients and the horizontal axis is *λ*

The ICC was computed by independently extracting radiomics features from a cohort of 20 patients by two physicians individually. In order to conduct a more comprehensive analysis, only the features with an ICC ≥ 0.800 were selected (Fig. 2). As a consequence, a total of 638 radiomics features were included as stable parameters for further data analysis. The use of LASSO regression (Fig. 3a, b) resulted in the identification of 10 radiomics features that exhibited non-zero coefficients. Table 2 presents the features together with their corresponding coefficients. The computation procedure of the Rad-score is as follows:

$$\mathrm{Rad}\;-\mathrm{score}\;=\;-0.049425808927957914\ast\mathrm{original}\_\mathrm{glcm}\_\mathrm{SumSquares}\;+\;0.0004907397303994439\ast\;\mathrm{original}\_\mathrm{gldm}\_\mathrm{DependenceVariance}-0.019827470658414142\ast\mathrm{wavelet}-\mathrm{LLH}\_\mathrm{glcm}\_\mathrm{Correlation}+0.013632176896904461\ast\mathrm{wavelet}-\mathrm{LHL}\_\mathrm{firstorder}\_\mathrm{Skewness}+2.825543660824761\ast\mathrm{wavelet}-\mathrm{LHL}\_\mathrm{glcm}\_\mathrm{Idmm}+0.08243375745020494\ast\mathrm{wavelet}-\mathrm{LHH}\_\mathrm{firstorder}\_\mathrm{Median}-0.25874341673431617\ast\;\mathrm{wavelet}-\mathrm{LHH}\_\mathrm{glszm}\_\mathrm{SmallAreaEmphasis}-0.30220545197336646\ast\mathrm{wavelet}-\mathrm{HLH_ngtdm_Strength}+0.0959692764620937\ast\mathrm{wavelet}-\mathrm{HHL_glszm_GrayLevelVariance}-0.1539916017410022\ast\mathrm{wavelet}-\mathrm{LLL_glcm_Imc}2$$

**Table 2:** Radiomics features associated with OS selected by LASSO regression

**Radiomics features**	**Coefficients**
original_glcm_SumSquares	-0.049425808927957914
original_gldm_DependenceVariance	0.0004907397303994439
wavelet-LLH_glcm_Correlation	-0.019827470658414142
wavelet-LHL_firstorder_Skewness	0.013632176896904461
wavelet-LHL_glcm_Idmn	2.825543660824761
wavelet-LHH_firstorder_Median	0.08243375745020494
wavelet-LHH_glszm_SmallAreaEmphasis	-0.25874341673431617
wavelet-HLH_ngtdm_Strength	-0.30220545197336646
wavelet-HHL_glszm_GrayLevelVariance	0.0959692764620937
wavelet-LLL_glcm_Imc2	-0.1539916017410022

**Fig. 4:**
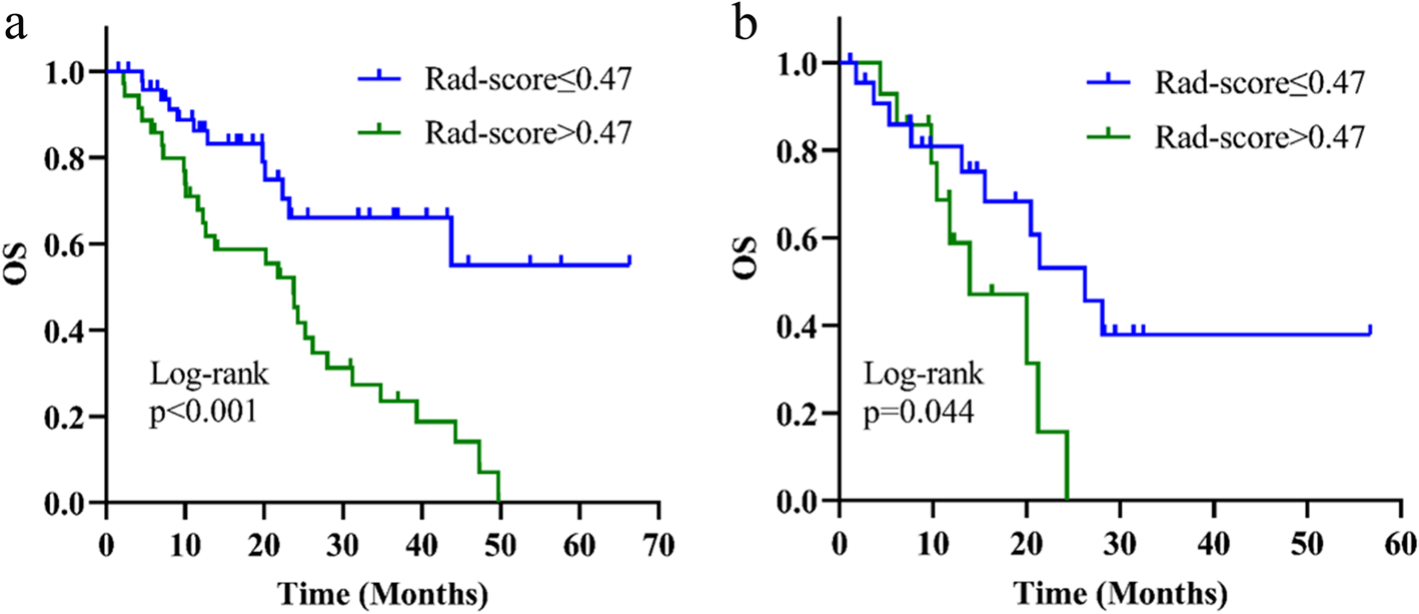
Kaplan–Meier survival curve based on Rad-score. **a** OS survival curve of patients in the training group: the green line represents patients with a high risk of mortality and the blue line represents patients with a low risk of mortality. The difference is significant between the two groups, *p* < 0.001. **b** OS survival curve of patients in the validation group: the green line represents patients with a high risk of mortality and the blue line represents patients with a low risk of mortality. The difference is significant between the two groups, *p* = 0.044

Patients were classified into two categories, high and low risk of mortality, based on the optimal cutoff value of Rad-score which was 0.47. Patients in the training group who had a Rad-score > 0.47 exhibited a considerably lower median OS compared to those with a Rad-score ≤ 0.47 (Fig. 4a, not reached vs 23.73 months, *p* < 0.001). The validation group yielded identical findings (Fig. 4b, 26.23 vs 13.93 months, *p* = 0.044).

### Development and validation of a predictive nomogram model

**Fig. 5:**
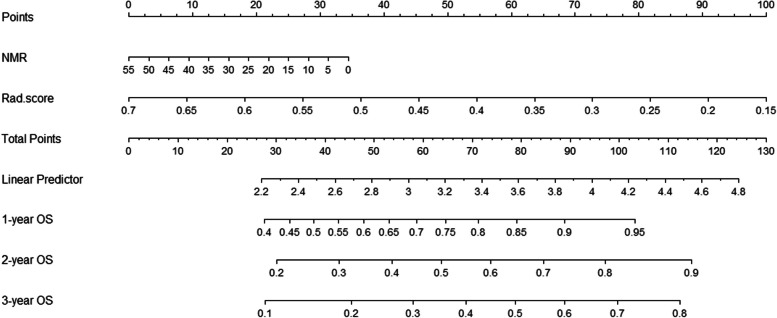
Nomogram model for predicting OS based on Rad-score

In order to develop a predictive model for OS, both univariate and multivariate analyses were performed to determine the predictors of it. The results of the univariate analysis (Table 3) indicated that there were statistically significant relationships between tumor location, NMR, and OS in both the training and validation cohorts. Table 4 displays the multivariate analysis results, which demonstrated that NMR and Rad-score were independent prognostic factors for OS. Based on the multivariate analysis, a nomogram model for predicting OS was developed (Fig. 5). As shown in Fig. 5, the probability of OS at 1, 2, and 3 years could be predicted for each patient based on NMR and Rad-score. In the training group, the nomogram model had a C-index of 0.619 (95% CI 0.518–0.720), while in the validation group, it was 0.573 (95% CI 0.385–0.760).

**Table 3:** Univariate analysis of prognostic factors associated with OS

**Variables**	**Training group**		**Validation group**	
	**HR (95% CI)**	***p***	**HR (95% CI)**	***p***
Gender (male vs female)	0.566 (0.236–1.356)	0.202	0.514 (0.167–1.582)	0.246
Age (≤ 65 vs > 65)	0.673 (0.359–1.260)	0.216	0.798 (0.322–1.975)	0.625
KPS (≤ 80 vs > 80)	1.162 (0.621–2.174)	0.638	0.404 (0.157–1.039)	0.060
Smoking status (never vs former/current)	1.161 (0.621–2.169)	0.641	1.621 (0.640–4.111)	0.309
Alcohol consumption (never vs former/current)	2.085 (1.115–3.896)	0.021	2.550 (0.954–6.811)	0.062
Tumor location (reference cervical)		0.040		0.011
Upper thoracic	0.588 (0.216–1.598)	0.298	0.117 (0.024–0.565)	0.008
Middle thoracic	0.314 (0.102–0.962)	0.043	0.212 (0.050–0.897)	0.035
Lower thoracic	1.337 (0.417–4.287)	0.625	0.839 (0.185–3.804)	0.820
Clinical stage (II vs III)	4.137 (1.264–13.543)	0.019	0.496 (0.174–1.412)	0.189
Radiation dose (reference > 60 Gy)		0.587		0.269
> 50.4 ≤ 60 Gy	1.027 (0.458–2.306)	0.948	2.220 (0.493–9.993)	0.299
≤ 50.4 Gy	1.574 (0.559–4.433)	0.391	0.913 (0.151–5.534)	0.922
Chemoradiotherapy (SCRT vs CCRT)	0.704 (0.373–1.327)	0.278	1.033 (0.414–2.575)	0.945
Radiation therapy (3D-CRT vs IMRT)	1.133 (0.517–2.481)	0.756	0.556 (0.182–1.702)	0.304
ALC (≤ 1.62 vs > 1.62 × 10^9^/L)	0.413 (0.218–0.783)	0.007	1.074 (0.429–2.678)	0.879
ANC (≤ 5.90 vs > 5.90 × 10^9^/L)	2.123 (1.030–4.376)	0.041	0.211 (0.028–1.586)	0.131
AMC (≤ 0.70 vs > 0.70 × 10^9^/L)	0.722 (0.299–1.743)	0.468	0.526 (0.152–1.826)	0.312
APC (≤ 214 vs > 214 × 10^9^/L)	0.601 (0.316–1.142)	0.120	0.565 (0.216–1.476)	0.244
NLR (≤ 2.01 vs > 2.01)	2.582 (1.278–5.217)	0.008	0.504 (0.197–1.289)	0.153
LMR (≤ 2.22 vs > 2.22)	0.351 (0.153–0.805)	0.013	1.619 (0.536–4.889)	0.393
PLR (≤ 137.64 vs > 137.64)	1.856 (0.967–3.559)	0.063	1.053 (0.397–2.789)	0.918
NMR (≤ 9.76 vs > 9.76)	3.020 (1.599–5.703)	0.001	3.803 (1.273–11.356)	0.017

*Abbreviations: KPS* Karnofsky Performance Status, *SCRT* sequential chemoradiotherapy, *CCRT* concurrent chemoradiotherapy, *IMRT* intensity modulated radiation therapy, *3D-CRT* 3-dimensional conformal radiation therapy, *ANC* absolute neutrophil count, *APC* absolute platelet count, *AMC* absolute monocyte count, *ALC* absolute lymphocyte count, *NLR* neutrophil-to-lymphocyte ratio, *NMR* neutrophil-to-monocyte ratio, *PLR* platelet-to-lymphocyte ratio, *LMR* lymphocyte-to-monocyte ratio

**Table 4:** Multivariate analysis of prognostic factors associated with OS

**Variables**	**Multivariate analysis**	
	**HR (95% CI)**	***p***
Tumor location (reference cervical)		0.062
Upper thoracic	0.500 (0.182–1.376)	0.180
Middle thoracic	0.317 (0.102–0.984)	0.047
Lower thoracic	1.146 (0.360–3.647)	0.818
NMR	2.457 (1.268–4.760)	0.008
Rad-score	2.388 (1.161–4.911)	0.018

**Fig. 6:**
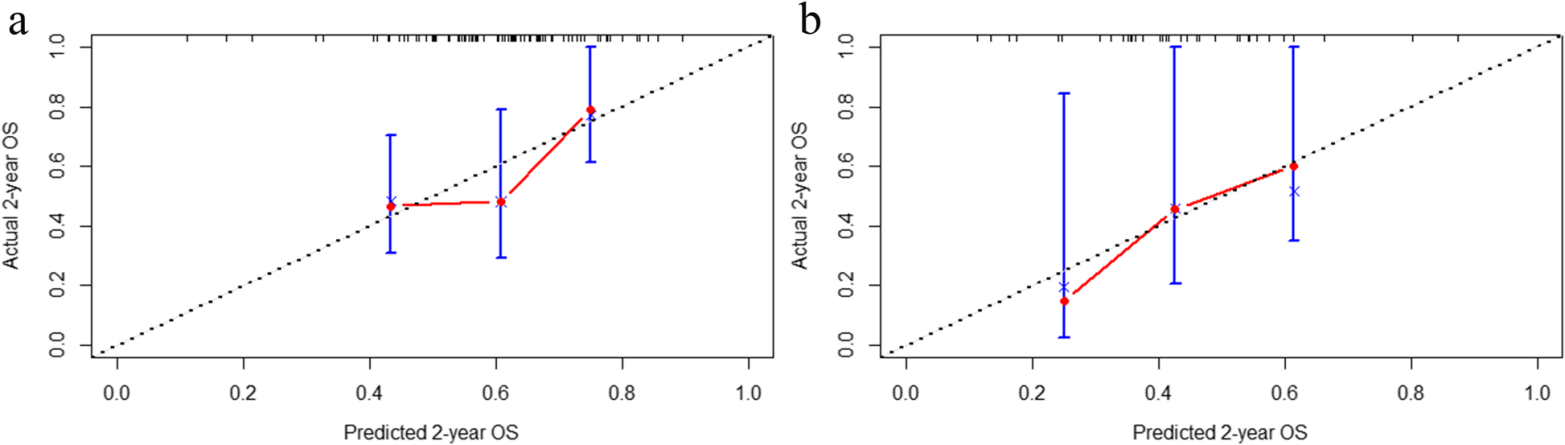
Calibration curve of the nomogram model in training group (**a**) and validation group (**b**). The *x*-axis represents the predicted 2-year OS, and the *y*-axis represents the actual 2-year OS. The black diagonal dashed line indicates the ideal nomogram and the red line indicates the observed nomogram. The more the calibration curve is close to the diagonal dashed line, the more agreement there is between the predicted results and the actual condition

A calibration curve was then developed to assess the accuracy of the nomogram. According to Fig. 6, the model accurately predicted the OS rate of 2 years, which closely matched the actual 2-year OS rate in both the training and validation groups.

## Discussion

In this research, we developed a nomogram model that integrated radiomics and hematological parameters through multivariate analysis in the training cohort. Next, we verified the accuracy of this model in a separate group of data. The findings indicated that Rad-score and NMR had the ability to categorize individuals into groups with either high or low risk of mortality. Using the model, we can initially calculate the likelihood of OS for every patient and pinpoint those who will gain greater advantages from dCRT.

Radiomics is a nascent methodology used in the analysis of medical pictures, whereby a diverse range of quantitative features are extracted from image data to assess the heterogeneity of malignancies. The aforementioned approach has significant relevance implications for personalized cancer treatment [23–25]. In their investigation, Gong et al. [26] used quantitative CT to develop a radiomics nomogram model to predict local recurrence-free survival in patients with ESCC who underwent dCRT. The research exhibited a considerable degree of predictive precision for the model. In a research conducted by Tang and colleagues [27], a prediction model for early recurrence of ESCC patients after trimodal therapy was created. All these studies demonstrated that radiomic features played a crucial part in assessing the local recurrence. Nevertheless, there is limited knowledge regarding the impact of radiomics on predicting OS in ESCC patients receiving dCRT. In our present study, a CT-based radiomics nomogram was established to predict the OS for these patients and was tested in a validation cohort. This research complements the existing proof of radiomics in predicting the risk of mortality for patients with ESSC who underwent dCRT.

Radiomics features extracted from tumor localization reflect the local tumor characteristics. Complementary to this, hematological parameters as an easily accessible index can reflect the overall condition of the organism. Furthermore, a substantial body of data is emerging that substantiates the involvement of inflammation in the initiation, progression, spread, and resistance to therapy of cancer [28, 29]. Several hematological markers, including the LMR, have been proposed as useful indicators of systemic inflammation and have been linked to the prognosis of several cancers [30–32]. According to a study done by Tang et al. [33], NMR has promise as a new and potentially valuable prediction tool for evaluating prognostic in individuals diagnosed with pancreatic cancer. Similar to the result of the study [33], in our present study, multivariate analysis suggested that pre-treatment NMR was the prognostic factor related to OS for ESCC patients after dCRT, and higher NMR was associated with inferior median OS. Currently, the mechanisms connecting increased NMR levels with worse survival rates in many cancers are poorly known. Multiple studies have shown that neutrophils produce substantial amounts of cytokines and chemokines such as vascular endothelial growth factor and matrix metalloproteinases. These substances play a significant role in facilitating angiogenesis, as well as fostering metastasis and the progression of tumors. In addition, it has been shown that it might have a suppressive impact on the immunological responses of natural killer cells and lymphocytes [34, 35]. Therefore, elevated ANC may be detrimental to survival, which was supported by the inverse association between neutrophil counts and OS in 1410 nasopharyngeal carcinoma patients [36]. At the same time, some studies have shown that an increased count of monocytes indicated a poor survival rate for solid tumors [29]. In our study, most patients had monocyte counts within the normal range. Therefore, the change in NMR was mainly attributed to an increase in ANC.

A nomogram was constructed for the prediction of 2-year OS by using Rad-score and NMR data via the use of a multivariate Cox regression model. The model’s C-index and calibration curves demonstrated strong predictive performance and accuracy in both the training and validation groups. Hence, it is our contention that the use of this Rad-score dependent predictive model may provide a more accurate means of forecasting OS, in a facilitative and affordable way.

Nevertheless, this study is bound by certain limitations. It is a retrospective analysis characterized by a somewhat limited sample size, which may explain the relatively low predictive accuracy of the model. Second, it is important to acknowledge that the research was done inside a singular institution, which might potentially limit the generalizability of the findings due to variations in CT scanners and scan settings at different establishments. In order to validate the dependability of these findings, more multicenter studies are necessary.

## Conclusion

The present work successfully devised and verified a prognostic model by using radiomics and hematological parameters, enabling the anticipation of OS in patients who have had dCRT. With this approach, doctors are able to quickly and accurately predict which patients with ESCC would respond favorably to dCRT.

## Data Availability

The datasets supporting the results reported in the article are available from the corresponding author upon reasonable request.

## Abbreviations

ALC: Absolute lymphocyte count
AMC: Absolute monocyte count
ANC: Absolute neutrophil count
APC: Absolute platelet count
dCRT: Definitive chemoradiotherapy
EC: Esophageal cancer
ESCC: Esophageal squamous cell carcinoma
ICC: Inter-class correlation coefficient
KPS: Karnofsky Performance Status
LASSO: Least absolute shrinkage and selection operator
LMR: Lymphocyte-to-monocyte ratio
NLR: Neutrophil-to-lymphocyte ratio
NMR: Neutrophil-to-monocyte ratio
OS: Overall survival
PLR: Platelet-to-lymphocyte
ROI: Region of interest
